# Key glycolytic branch influences mesocarp oil content in oil palm

**DOI:** 10.1038/s41598-017-10195-3

**Published:** 2017-08-29

**Authors:** Nurliyana Ruzlan, Yoke Sum Jaime Low, Wilonita Win, Noor Azizah Musa, Ai-Ling Ong, Fook-Tim Chew, David Appleton, Hirzun Mohd Yusof, Harikrishna Kulaveerasingam

**Affiliations:** 1 Biotechnology & Breeding Department, Sime Darby Plantation R&D Centre, Selangor, Malaysia; 2 Sime Darby Renewables, Sime Darby Plantation Sdn Bhd, Selangor, Malaysia; 3 Department of Biological Sciences, National University of Singapore, Singapore, Singapore

## Abstract

The fructose-1,6-bisphosphate aldolase catalyzed glycolysis branch that forms dihydroxyacetone phosphate and glyceraldehyde-3-phosphate was identified as a key driver of increased oil synthesis in oil palm and was validated in *Saccharomyces cerevisiae*. Reduction in triose phosphate isomerase (TPI) activity in a yeast knockdown mutant resulted in 19% increase in lipid content, while yeast strains overexpressing oil palm fructose-1,6-bisphosphate aldolase (*EgFBA*) and glycerol-3-phosphate dehydrogenase (*EgG3PDH*) showed increased lipid content by 16% and 21%, respectively. Genetic association analysis on oil palm SNPs of *EgTPI* SD_SNP_000035801 and *EgGAPDH* SD_SNP_000041011 showed that palms harboring homozygous GG in *EgTPI and* heterozygous AG in *EgGAPDH* exhibited higher mesocarp oil content based on dry weight. In addition, AG genotype of the SNP of *EgG3PDH* SD_SNP_000008411 was associated with higher mean mesocarp oil content, whereas GG genotype of the *EgFBA* SNP SD_SNP_000007765 was favourable. Additive effects were observed with a combination of favourable alleles in TPI and FBA in Nigerian x AVROS population (family F7) with highest allele frequency GG.GG being associated with a mean increase of 3.77% (p value = 2.3E^−16^) oil content over the Family 1. An analogous effect was observed in yeast, where overexpressed *EgFBA* in TPI^*-*^ resulted in a 30% oil increment. These results provide insights into flux balances in glycolysis leading to higher yield in mesocarp oil-producing fruit.

## Introduction

The oil palm (*Elaeis guineensis* Jacq.) is the highest yielding oil crop and has therefore become extremely important for food supply^1^. Increasing oil yield per hectare is a major goal to meet growing demand for edible oil and biodiesel without the need for increased agriculture land. However, oil yield is genetically a complex trait involving many genes, especially for a perennial oil fruit plant such as oil palm. Therefore, increasing yield through traditional breeding is relatively slow.

The glycolytic pathway has been proposed as one of the key biosynthetic steps for supplying precursors and controlling the rate of oil biosynthesis in oil palm mesocarp tissue based on previous studies^2^. Higher protein expression level of fructose-1,6-bisphosphate aldolase (FBA) combined with a reduced level of triose phosphate isomerase (TPI) and glyceraldehyde-3-phosphate dehydrogenase (GAPDH) with associated metabolite concentration changes in high-yielding oil palm plants suggested important flux balance changes in glycolysis are closely linked with oil yield^3^. In particular, Teh and co-workers^4^ noted an apparent divergence of carbon flux towards glycerol-3-phosphate (G3P) preceding and during lipid biosynthesis in the fruit of high-yielding palms. Transcript levels of G3PDH in the mesocarp has also been shown to be higher during fruit ripening and oil biosynthesis^5^.

FBA catalyzes the conversion of fructose-1,6-bisphosphate (FBP) into triose phosphates, dihydroxyacetone phosphate (DHAP) and glyceraldehyde-3-phosphate (GAP). It has been shown to play a significant role in seed oil biosynthesis, being highly expressed in high oil yield cultivars of soybean, *Camellia oleifera*, and rapeseed^6–8^. TPI subsequently catalyzes the inter-conversion of DHAP and GAP to establish a carefully controlled balance of flux thereafter^9^. Studies in yeast, *Arabidopsis thaliana* and canola have demonstrated that reduced TPI activity causes a shift in glycolytic flux towards glycerol and leads to increased oil yield^10–12^. Downstream of the FBA/TPI branch in glycolysis, G3PDH is also known to play a major role in supply of glycerol in the form of G3P to support neutral lipid biosynthesis^13, 14^. Increased G3P levels in *Brassica napus* affect the rate of triacylglycerol (TAG) formation and resulted in a 40% increase in lipid content^15^.

Our Group previously found differential in gene expression as well as protein and metabolite levels in glycolysis pathway were associated with mesocarp oil content^3, 4, 16^. In this study, we validated the oil palm glycolytic gene function through loss-of-function mutants in *Saccharomyces cerevisiae* as a model system. We also have identified SNPs on the glycolytic genes significantly associated with mesocarp oil content (oil to dry mesocarp percentage or O/DM) in selected oil palm populations. Epistasis analysis between TPI and FBA was conducted to investigate their combination effect towards altering flux at the key glycolysis branch point and its impact on oil biosynthesis and content in oil palm, in addition to their utility as selection markers in breeding.

## Results

### Validation of oil palm glycolytic genes in yeast

**Figure 1:**
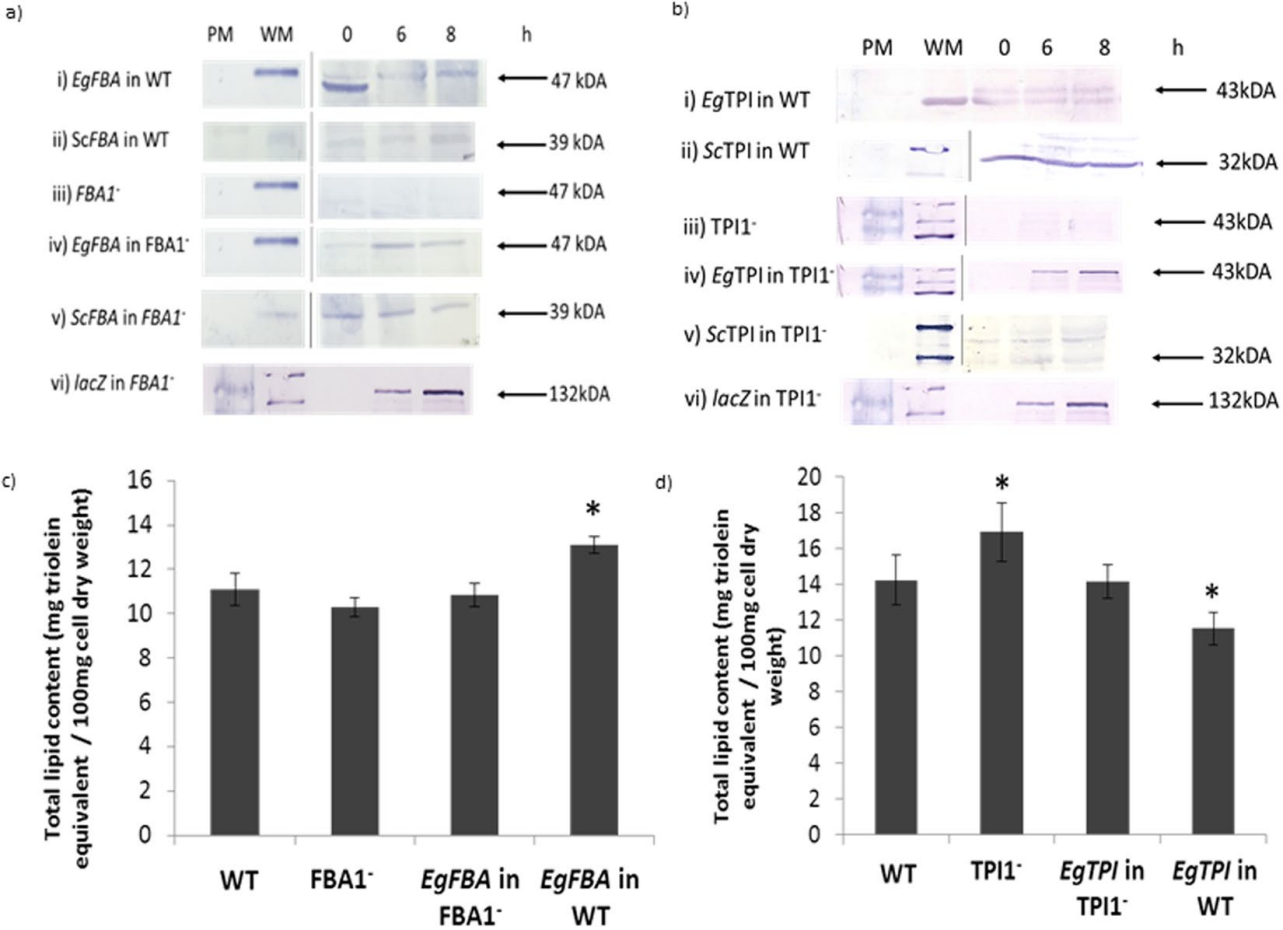
Expression of oil palm fructose-1,6-bisphophaste aldolase (*EgFBA*) and triose phosphate isomerase (*EgTPI*) using *Saccharomyces cerevisiae*. (**a**) Verification on *EgFBA* transformants was conducted using Western Blot assay with anti-FBA antibody (1:50000). (ai) Protein of *EgFBA* was detected at 47 kDa for overexpression of *EgFBA* in WT at 6 and 8 hours after induction with galactose. Lower size protein bands were observed at 0hr due to non-specific binding of the anti-FBA antibody. (aii) *ScFBA* was also observed at 39 kDa in the WT strain. (aiii and aiv) The function of *EgFBA* was validated as the protein band of *EgFBA* was observed in *EgFBA* complemented in FBA1^−^. This was further confirmed as no protein band was observed at 47 kDa in the FBA1^−^ strain. (av) Similarly, *ScFBA* band at 39 kDa was also observed in the FBA1^−^ indicating the presence of *ScFBA* in heterozygous knock out strain. (avi) lacZ in FBA1^−^ serve as positive control for the assay. Presence of the 132 Da band indicated presence of pYES 2.1 TOPO vector in TPI1^−^. (**b**) Validation of palm transformants using the anti-TPI antibody (1:500). Presence of the 132 Da band indicated presence of pYES 2.1 TOPO vector in FBA1^−^. (bi) Protein of *EgTPI* was detected at 43 kDa for overexpression of *EgTPI* in WT at 6 and 8 hours after induction with galactose. Lower size protein bands were observed due to non-specific binding of the anti-TPI antibody. (bii) *ScTPI* was also observed at 32 kDa in the WT strain. (biii and biv) The function of *EgTPI* was validated as the protein band of *EgTPI* was observed in *EgTPI* complemented in TPI1^−^. This was further confirmed as no protein band was observed at 43 kDa in the TPI1^−^ strain. (bv) Similarly, *ScTPI* band at 32 kDa was also observed in the TPI1^−^ indicating the presence of *ScTPI* in heterozygous knock out TPI1^−^ strain. (bvi) lacZ in TPI1^−^ serve as positive control for the assay. Presence of the 132 Da band indicated presence of pYES 2.1 TOPO vector in TPI1^−^. PM and WM refer to protein marker and western marker used as ruler. (**c**,**d**) Effect of expression of *EgFBA* and *EgTPI* in their respective mutants and WT on yeast lipid content. The data presented are the mean ± standard deviation (n = 4–6), *p < 0.05 to the WT. All the blots photos are cropped for display.

Oil palm glycolytic gene isolation, function and impact on lipid biosynthesis were validated using complementation and overexpression in *Saccharomyces cerevisiae*. Four key genes directly involved in the glycolytic branch, triose phosphate isomerase (*EgTPI)*, fructose-1,6-bisphosphate aldolase (*EgFBA*), glyceraldehyde-3-phosphate dehydrogenase (*EgGAPDH*) and glycerol-3-phosphate dehydrogenase (*EgG3PDH*), were isolated from the mesocarp tissue of oil palm and transformed into yeast (Fig. 1, Supplementary Figures 1–7). All four genes were successfully cloned and expressed into the yeast wild type (WT) and mutant strains, as confirmed by yeast colony PCR and Western blot assay of total protein extracts. In all cases, comparable lipid content to wild type was achieved when analogous oil palm genes were expressed in yeast knock out mutants. Lipid content in a heterozygous yeast mutant lacking in FBA was slightly lower than WT (Fig. 1c). It was observed that complementation of *EgFBA* into FBA1^−^ mutant restored lipid content to levels similar to WT. Overexpression of *EgFBA* in WT further increased lipid content by 18% compared to WT. Lipid content of yeast TPI knockdown mutant was about 19% higher compared to WT, while complementing *EgTPI* into TPI1^−^ mutant led to a reduction in lipid content (Fig. 1d). Over-expression of *EgTPI* in WT further reduced lipid content in the yeast transformant compared to WT. Functional complementation of *EgG3PDH* into yeast mutant lacking G3PDH (GPD1^−^) increased lipid content and rescued the osmosensitive phenotype of the GPD1^−^ mutant (Supplementary Figure 4). Further overexpression of *EgG3PDH* led to an increase of lipid content of 21% over WT. Similar to TPI1^−^ mutant, yeast mutant lacking GAPDH (TDH3^−^) exhibited a significant increase (p < 0.01) in lipid content compared to WT, while complementing *EgGAPDH* restored lipid content to levels similar to WT (Supplementary Figure 6).

### Association analysis of glycolytic genes

Genome wide association study (GWAS) was previously conducted on a population of 2,045 palms with 7 years of phenotype data to find alleles that were associated with high O/DM from breeding crosses between Deli *Dura* and Avros or Dumpy AVROS *Pisifera* palms^17^. A population of Nigerian AVROS (n = 586 palms) palms resulting from crosses between semi-wild Nigerian *Dura* and AVROS *Pisifera* was also included in the analysis as a reference. We mined this dataset for association of SNPs on the key glycolytic genes (summary shown in Table 1). Successfully genotyped SNPs are listed in the table with minor and major alleles, minor allele frequency (MAF) and p-value information. We identified 14 SNPs on the four genes of interest where the location of each SNP on the genes was determined with verified gene sequences and organization based on the published oil palm physical map. Predicted gene function changes were made based on the SNP locations within gene regions; promoter SNPs as potential transcription factor binding site effects, intronic SNPs for exon splicing or intron retention effects, exonic SNPs for any amino acid changes, or 3′ UTR region SNPs for microRNA binding site changes (Supplementary Table 1 and 2).

**Table 1:** Successfully genotyped SNPs for fructose-1,6-bisphosphate aldolase (*EgFBA*), triose phosphate isomerase (*EgTPI*), glycerol-3-phosphate dehydrogenase (*EgG3PDH*) and glyceraldehyde-3-phosphate dehydrogenase (*EgGAPDH*) for association data analysis. MAF stands for Minor Allele Frequency.

SNP ID	Chr	Position (bp)	Nigerian x AVROS				Deli x AVROS				Deli x AVROS			
			Minor allele	Major allele	MAF	p value	Minor allele	Major allele	MAF	p value	Minor allele	Major allele	MAF	p value
***EgFBA***														
SD_SNP_000007765	2	24881326	A	G	0.338	0.011	A	G	0.175	0.223	A	G	0.162	0.552
SD_SNP_000007766	2	24879331	G	A	0.451	0.425	G	A	0.242	0.005	G	A	0.285	0.105
***EgTPI***														
SD_SNP_000151220	16	19626161	T	C	0.372	0.112	T	C	0.381	0.062	C	T	0.369	0.206
SD_SNP_000035800	16	19621888	A	G	0.034	0.613	A	G	0.342	0.233	A	G	0.320	0.287
SD_SNP_000035801	16	19620304	A	G	0.406	**0.015**	A	G	0.481	0.574	A	G	0.411	0.780
SD_SNP_000035802	16	19619353	G	A	0.185	**0.027**	G	A	0.286	0.549	G	A	0.274	0.747
SD_SNP_000035803	16	19615813	G	A	0.230	0.153	G	A	0.383	0.728	G	A	0.354	0.999
***E***_***g***_ ***G3PDH***														
SD_SNP_000008411*	5	26031150	A	G	0.005	0.100	A	G	0.215	0.155	A	G	0.265	**0.032**
***E***_***g***_ ***GAPDH***														
SD_SNP_000041010	9	21239379	C	A	0.2966	0.097	C	A	0.1195	0.721	C	A	0.0561	0.117
SD_SNP_000041011	9	21235385	A	G	0.3985	0.289	A	G	0.2375	**0.001**	A	G	0.2808	0.796
SD_SNP_000041012	9	21231353	A	G	0.3055	**0.004**	G	A	0.4292	0.270	G	A	0.3670	0.335
SD_SNP_000041013	9	21229798	C	A	0.3487	0.832	C	A	0.1276	0.818	C	A	0.0592	0.065

*SD_SNP_000008411– Homo for Nigerian x AVROS.

**Figure 2:**
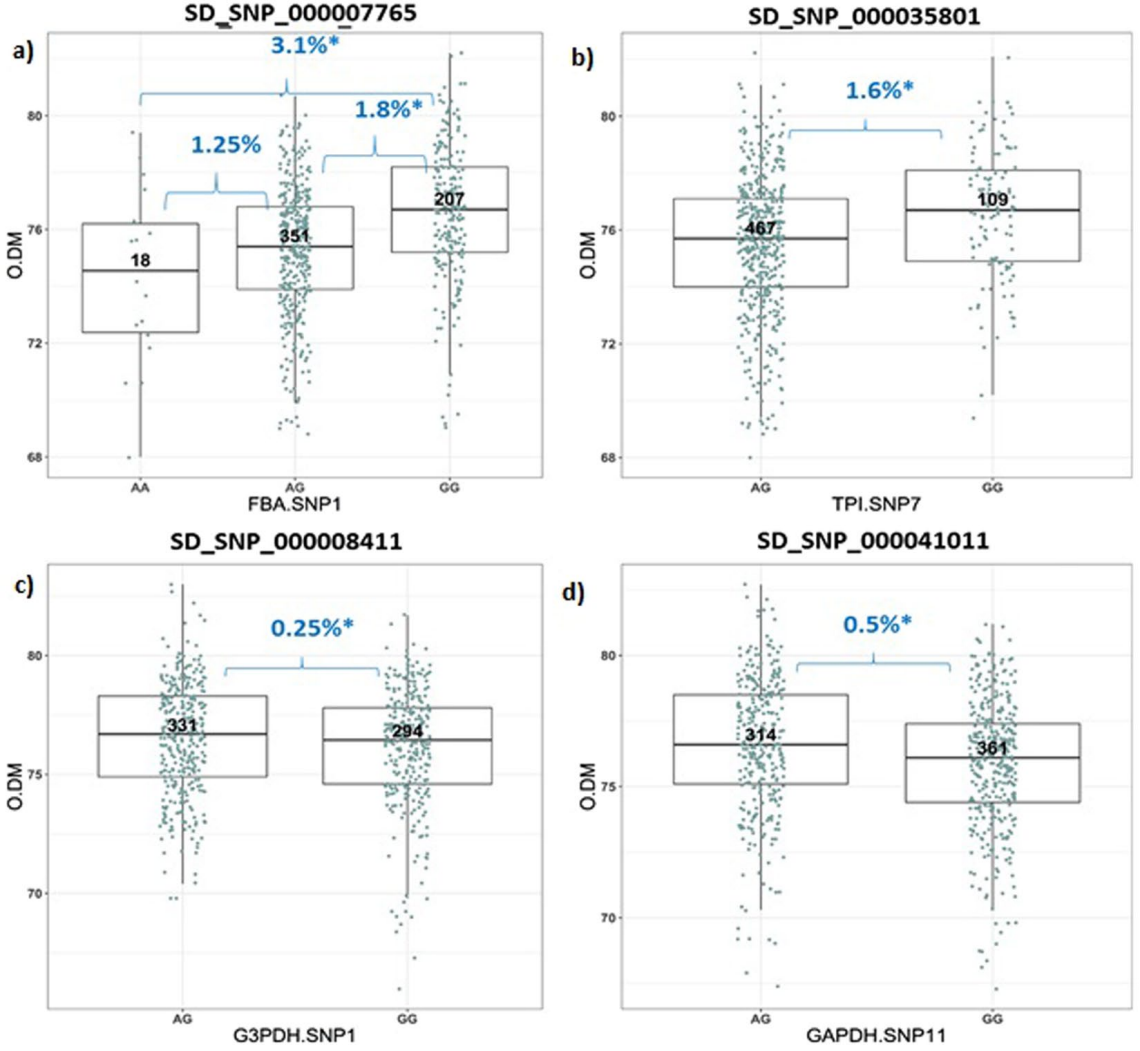
Trait association data for significant SNPs in selected population for glycolytic genes (**a**) Trait association data for oil palm genotypes against oil to dry mesocarp (O/DM) value for *EgFBA* SNP SD_SNP_000007765. Approximately, 207 individuals harboring genotype homozygous GG the highest O/DM value in Nigerian x AVROS (n = 576 individual palms). (**b**) Boxplot showing association of the oil palm genotype against the oil to dry mesocarp (O/DM) value for *EgTPI* for SD_SNP_000035801. Approximately, 109 individuals harboring genotype homozygous GG had the highest O/DM value in Nigerian x AVROS (n = 576 individual palms). (**c**) Boxplot showing the association data for *EgG3PDH* SNP SD_SNP_000008411 in Deli Dura x AVROS where 331 individuals harboring heterozygous genotype AG had higher O/DM value (n = 625 individual palms). (**d**) Boxplot showing the association data for *EgGAPDH* SNP SD_SNP_000041011. Approximately, 314 individuals harboring genotype heterozygous AG had the highest O/DM value in Deli Dura x Dumpy AVROS (n = 675 individual palms). The data presented are the mean ± standard deviation, *p < 0.05 to the WT.

*EgFBA* intronic SNP SD_SNP_000007765 association was found to be significant within Nigerian x AVROS population (n = 576 palms) with homozygous GG palms found to have a mean O/DM of 77.1% compared to 75.3% for AG-genotype palms (Fig. 2a), p-value = 0.01. *EgTPI* intronic SNP, SD_SNP_000035801 was found to be significant in the same population (n = 576 palms, p-value = 0.015). Palms with homozygous GG (n = 109) were found to have a mean O/DM of 77.2% compared to 75.6% for those with heterozygous AG genotype (n = 467) as shown in Fig. 2b. *EgG3PDH* promoter SNP, SD_SNP_000008411 was found to be significantly associated in the Deli x AVROS population (n = 625 palms) where palms carrying heterozygous AG genotype (n = 331) were found to have a higher mean O/DM of 76.7% compared to homozygous GG, 76.45% (n = 294), p-value = 0.032 (Fig. 2c). For *EgGAPDH*, only intronic SNP SD_SNP_000041011 was found to be associated to O/DM in the Deli x Dumpy AVROS population (n = 675 palms) with p-value = 0.001. Heterozygous AG (n = 314) genotypes had a higher mean of 76.6% O/DM compared to homozygous GG (n = 361) with mean of 76.1% as shown in Fig. 2d.

### Interaction of SNPs associated with oil content in Nigerian x AVROS population

**Figure 3:**
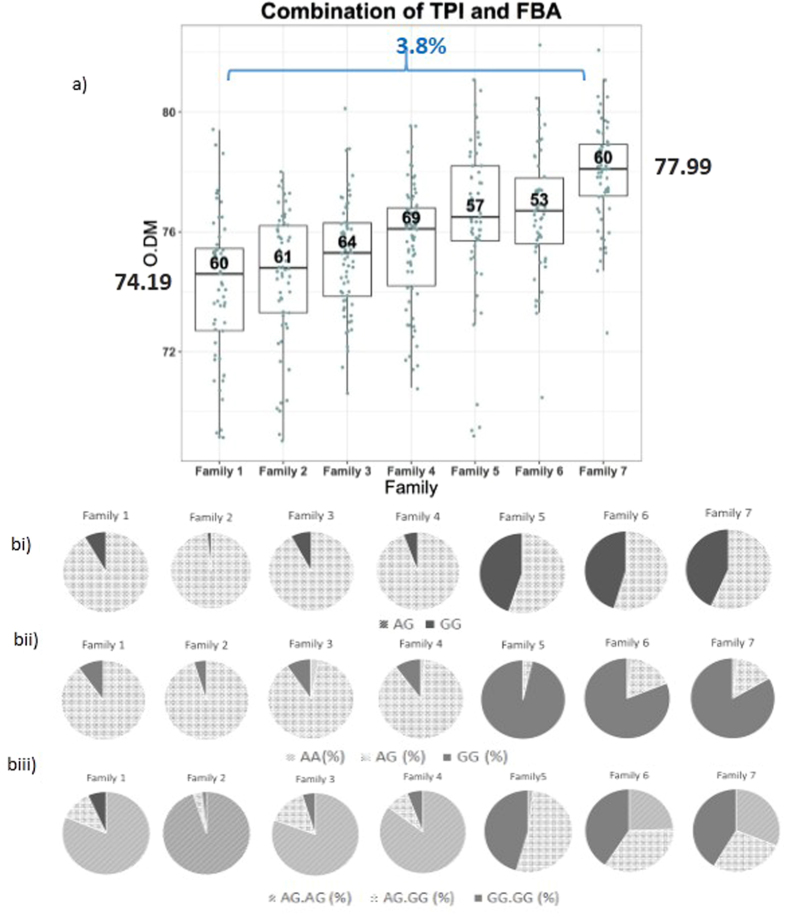
SNP combination analysis for *EgTPI* SD_SNP_000035801 and *EgFBA* SD_SNP_000007765 on oil to dry mesocarp (O/DM) in Nigerian x AVROS. (**a**) Boxplot showing the association data for selected families of Nigerian X AVROS where Family 7 had the highest mean mesocarp oil content. bi) Pie charts represent the percentage allele frequency in *EgTPI* SD_SNP_000035801 and *EgFBA* SD_SNP_000007765. *EgTPI* SD_SNP_000035801 had two genotypes; AG and GG where Family 7 possessed the highest percentage of allele frequency GG and Family 1 had highest percentage of allele frequency of heterozygous AG. (**bii**) Similar finding was observed for *EgFBA*, where Family 7 had higher percentage of allele frequency GG. Individuals harboring genotype AA were uncommon. (**biii**) Pie charts showing the combination of genotypes for both SNPs, where Family 7 with higher percentage of allele frequency GG.GG had highest O/DM value (n = 429 individual palms).

**Figure 4:**
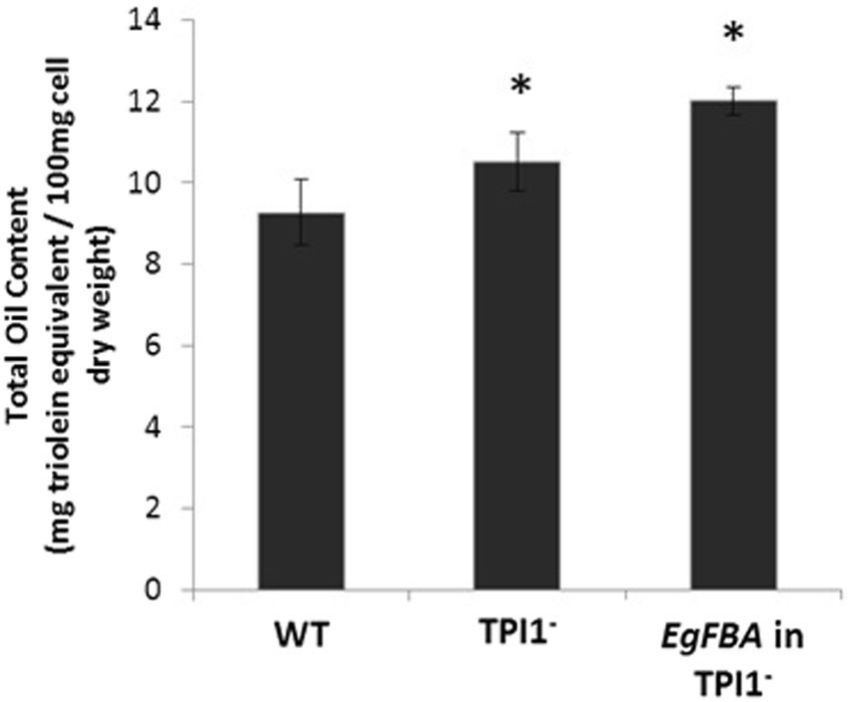
Combination effect of *EgFBA* and TPI1^−^ strain on oil accumulation in yeast. Lipid content in strain with overexpressed *EgFBA* in TPI1^−^ mutant and WT at late exponential phase. The data presented are the mean ± standard deviation (n = 4–6), *p < 0.05 compared to WT.

Interaction analysis of the two significant SNPs in *EgTPI* (SD_SNP_000035801) and *EgFBA* (SD_SNP_000007765) was conducted to investigate potential synergistic effects. The Nigerian x AVROS population was divided into seven families based on their genotype backgrounds using Principle Component Analysis (Supplementary Figure 8). The remaining 152 palms were excluded from the analysis due to low sample size after segregation into families. Based on the distribution of genotypes within the families, it was observed that family with highest percentage of homozygous GG for each of the TPI and FBA SNPs (Family 7) had highest mean O/DM. Whereas, family containing a highest percentage of palms harboring A allele (Family 1) had lowest mean mesocarp oil content by as much as 3.8% (Fig. 3a, bi and bii). Combination of *EgTPI* (SD_SNP_000035801) and *EgFBA* (SD_SNP_000007765) SNPs, Family 7 had the highest allele frequency GG.GG along with the highest mean mesocarp oil content (Figure 3biii). Interestingly, another experiment using yeast mutant strains that investigated the reduction of TPI activity concurrent with up-regulation of EgFBA, as proposed in earlier studies^3, 4^ demonstrated that overexpression of EgFBA in TPI1^−^ yeast mutant further enhanced lipid accumulation by 30% (Fig. 4).

## Discussion

A previous study by our Group revealed that gene expression, along with protein and metabolite levels in the glycolysis pathway were associated with oil content in oil palm fruit mesocarp during fruit development^3, 4, 16^. In particular, the differential results suggested a divergence in carbon flux at the glycolytic branch point involving the set of four enzymes: FBI, TPI, GAPDH and G3PDH. Results from these studies suggested an overall higher carbon flux through this branch point as evidenced by higher abundance of FBA protein at 16 weeks after pollination (WAP). The up-regulation of FBA in high-yielding palms is likely to increase flux through glycolysis into *de novo* fatty acid synthesis^18^. In addition, metabolite levels downstream after the branch indicated an alteration of flux towards glycerol production from lower TPI expression. Metabolite comparisons between high-yielding and low-yielding palms showed glycerol-3-phosphate levels were 43% higher in high-yielding mesocarp at 16 WAP while downstream of GAP, 3-phosphoglyceric acid levels were 26% lower^4^. Protein levels of TPI and GAPDH were also lower in high-yielding oil palms and could potentially be related to this observed flux balance change. It was also expected that the activity of G3PDH would also impact the flux balance of this branch. These observations in oil palm presented an intriguing opportunity to investigate the concerted interaction of these genes as a driver of oil biosynthesis in mesocarp tissue.

This present study represents the first reported validation of oil palm glycolytic gene function using a single cell eukaryote, *Saccharomyces cerevisiae* as a host organism for heterologous expression of proteins. Overexpression of FBA in WT yeast led to a significant increase in lipid content over the WT yeast, while a knockdown of FBA activity in a mutant strain had the opposite effect on lipid accumulation. Similarly, FBA appeared up-regulated in high-yielding palms at the onset of fruit ripening and lipid accumulation. FBA is an important metabolic enzyme in the glycolytic pathway, producing key metabolites for oil biosynthesis and cell metabolism^19, 20^. A yeast TPI1^−^ mutant, exhibited an increase in lipid content of approximately 19% compared to WT, while a reduction of lipid content was observed when *EgTPI* was overexpressed in yeast cells. These results are concordant with other reports, whereby a severe reduction of TPI activity led to an accumulation of lipids in plant and human cells^11, 21, 22^. Reduced TPI activity is known to cause alteration in glucose metabolism that leads to elevation of DHAP and G3P in yeast^23^. These metabolites serve as important carbon precursors for lipid biosynthesis. The third gene investigated, G3PDH plays a major role in the biosynthesis of glycerol and neutral lipid through reduction of DHAP into G3P in yeast, *Brassica napus*, and *Phaeodactylum tricornutum* ^13–15, 24, 25^. The results in this present study are consistent with findings from previous approaches used to increase oilseed in other oil crops, which have shown higher expression or activity of *EgG3PDH* leads to an increase in total oil content^7, 14^. It has been reported that increased G3P levels in *Brassica napus* increases the rate of TAG formation and results in a 40% increase in lipid content^15^. The transcript level of G3PDH in oil palm mesocarp was 1.7-fold higher during ripening and oil production stages^5^, demonstrating carbon flux through glycolysis to producing more G3P is important for lipid production in oil palm mesocarp^26^. Downstream of FBA and TPI, overexpression of *EgGAPDH* in WT yeast resulted in a decrease in lipid content. The results shown are in concordance with protein level observations in oil palm using differential gel electrophoresis (DIGE), which indicated that GAPDH was down-regulated in high-yielding palms^3^. Again, these findings are consistent with observations in sunflower where TPI and GAPDH activities were found to be higher in the low-oil content sunflower lines^27^. GAPDH is important for the maintenance of cellular ATP levels, carbohydrate metabolism, TCA cycle and amino acid synthesis. Transcriptome analysis of the phosphorylating GAPDH null mutant in *Arabidopsis thaliana* showed some glycolytic and the tricarboxylic acid (TCA) cycle enzymes involved in carbon flux decreased^28^. Overall, the function of the isolated oil palm glycolytic genes was confirmed in the yeast system and alteration of expression appeared to concur with biochemical comparisons between high- and low-yielding oil palms. Furthermore, we observed a 30% increase in lipid content in a TPI^-^ mutant *S. cerevisiae* strain with overexpressed *EgFBA* compared to wild type indicating possible synergistic effects. The significant increase in lipid accumulation is suggestive of the key role that the flux balance through this glycolytic branch plays for oil biosynthesis in both the yeast and oil palm systems.

### SNP association with oil content

Genetic variance caused by nucleotide changes in gene sequence will affect gene function and could be important for increasing oil production in oil palm. The previous biochemical analysis of high-yielding oil palms and now validation of the gene function of oil palm glycolytic genes presented above led us to investigate the association of SNPs on *EgFBA*, *EgTPI, EgG3PDH and EgGAPDH* with oil content in larger oil palm populations. Genetic association analysis identified one SNP each on each glycolytic genes (SD_SNP_000007765, SD_SNP_000035801, SD_SNP_000008411 and SD_SNP_000041011) to be significantly associated with mesocarp oil content (O/DM) with p-values less than 0.05 in different population.

At the gene function level, we need to understand how gene variance caused changes in gene function. SNP effects on gene function were predicted based on the location of the SNPs. Analysis of intronic SNP *EgFBA* SD_SNP_000007765 showed that nucleotide changes have splicing effect potential by exon removal where the FBA domain and active site is located, and this may produce dysfunctional *EgFBA* protein activity for one of the genotypes. *EgTPI* SNP SD_SNP_000035801 was also predicted to cause splicing by removal of exon 3. We further analyzed the effect of exon 3 splicing on *EgTPI* protein structure and found that it would result in substrate binding site loss. Promoter *EgG3PDH* SNP SD_SNP_000008411, was predicted to have no change in the promoter binding site, hence the trait association of the SNP may be caused by another SNP within the same linkage disequilibrium block. Gene function effect prediction of intronic *EgGAPDH* SNP SD_SNP_000041011 indicated potential exon splicing that may affect changes in the protein structure. Validation of SNP effects will require *in-vitro* functional investigation of the effect of the SNP nucleotide changes. Associated SNPs identified in this study may fall into linkage disequilibrium with causal SNPs in certain population groups. Therefore re-sequencing of the four genes using more individuals in carefully selected populations will enable the discovery of additional SNPs and probe the linkage disequilibrium blocks further. This will provide us with more information and insights into the association of these glycolytic genes with oil content and reveal candidate causal sequence variants for observed gene expression and activity differences.

### Combining analysis of significant SNPs in Nigerian x PORIM

Oil biosynthesis in plants is the result of complex biosynthetic pathways, controlled by many genes in combination. Many of the genes have small effects, and a few are strongly associated with oil yield^29^. In some cases multiple genes exhibit synergistic effects on the trait. Previous biochemical studies of high- and low-yielding oil palms, and the yeast validation experiments presented here strongly suggested synergy between *EgFBA* and *EgTPI*. Genetic association analysis suggested effects of variation in these genes was strongest in the Nigerian x AVROS population because both SNPs, SD_SNP_000035801 and SD_SNP_000007765 are significantly associated in this population. The allele frequency for GG of both SNPs was found higher in Nigerian x AVROS as compared to other populations. Genotype analysis of the families in Nigerian x AVROS based on mesocarp oil content clearly showed that Family 7 that had highest percentage of genotype GG for both SNPs, *EgTPI* and *EgFBA* along with higher O/DM. The mean mesocarp oil content for Family 7 was 77.99%, 3.8% higher than the mean mesocarp oil content for Family 1; 74.19% (p-value = 2.29 × 10^−16^). Single SNP analysis for both SD_SNP_000035801 and SD_SNP_000007765 with O/DM revealed that families with higher frequency of palms carrying genotype G will have higher mean mesocarp oil content than families with lower frequency of genotype G. For example in *EgTPI* SD_SNP_000035801, it was observed that Family 7 with 43.33% homozygous GG had higher O/DM than Family 1 with 8.33% homozygous GG. A similar finding was also observed for *EgFBA* SD_SNP_000007765 where Family 7 had a higher proportion of palms carrying homozygous GG (83.33%) than Family 1 with lower proportion of homozygous GG (10%). Combination analysis of both SNPs revealed that Family 7 with highest mean mesocarp oil content had the highest frequency of GG.GG (44.07%) as compared to Family 1 with lowest GG.GG percentage (6%). These data suggests that polymorphism in *EgFBA* and *EgTPI* contributes toward the determination of palms with higher yield within specific populations. In future, identification and validation of the causal variants will be required in order to understand the foundation and the mechanism by which they contribute to increased oil biosynthesis. Overall, the combined genetic association data further confirms the significance of these genes for the control of oil biosynthesis.

In addition, these SNPs may be used as a screening tool for selection of high-yielding palms, in particular in wild populations. Mesocarp oil content is one of the more heritable oil palm traits and contributes significantly to total oil yield^32^. The SNPs in this study could be used as a marker assisted selection tool (MAS) to enable plant breeders to identify and propagate palms with highest oil content without the need for laborious fruit analysis to be conducted These SNPs can also be used together with other marker sets to combine desirable traits and for genomic selection. Application of MAS in oil palm can significantly expedite the breeding program^17, 32^.

## Conclusions

This study provides further evidence into the significant concerted role that the genes in the glycolysis branch play for oil biosynthesis in oil palm mesocarp tissue, as well as in a yeast model system. Overexpression of *EgFBA* in *S. cerevisiae* led to higher lipid accumulation and suggests this is a key rate controlling step in glycolysis. While, reduced expression of TPI and GAPDH or overexpression of *EgG3PDH* in yeast also resulted in significant increase in lipid accumulation, presumably by directing more flux towards the lipid backbone, glycerol. A combination of overexpressed *EgFBA* in a TPI1^−^ mutant resulted in an even larger increase in lipid accumulation of 30%. Genetic association analysis for mesocarp oil content in oil palm identified four significantly associated of SNPs on the four genes of interest: *EgTPI* (SD_SNP_000035801), *EgFBA* (SD_SNP_000007765), *EgGAPDH* (SD_SNP_000041011) and *EgG3PDH* (SD_SNP_000008411). We have also successfully identified palms harboring specific genotypes for each of the SNPs that record higher oil yield in field trials. In addition, significant additive effects were identified between and *EgTPI* SD_SNP_000035801 and *EgFBA* SD_SNP_000007765 supporting the hypothesis of the concerted effect these genes have on directing flux and controlling oil biosynthetic rate. Further fine-mapping and larger scale genotyping of selected oil palm populations will enable greater confidence on the identification of causal SNPs and provide a foundation for functional validation of their mechanisms. However, these SNPs can already provide insight into selection for breeding in advanced and wild populations of oil palm towards optimized oil production.

## Methods

### Functional validation of oil palm glycolytic genes using *Saccharomyces cerevisiae* as a model system

The sequences of the genes were obtained using the published oil genome browser by Malaysian Palm Oil Board (http://gbrowse.mpob.gov.my/fgb2/gbrowse/Eg5/) based on homology with other plant species eg; *Arabidopsis thaliana* and *Oryza sativa*. Specific primers were designed to amplify the full length gene or open reading frame (ORF) by PCR using 100 ng/µL of pool cDNA of previously identified high-and low-yielding palm fruits at 12, 16, 20 and 22 weeks after pollination (WAP) as a template. Amplification was performed for 5 minutes at 94 °C followed by 35 cycles of 30sec-1min at 94 °C, 30 seconds at 50–60 °C (depending on the primer set), 1–2 minutes at 72 °C and final extension for 5 minutes at 72 °C. Expected PCR products were checked on 2% agarose gel and later cloned into pGEM-T Easy vector (Promega Corporation, Madison) before the plasmid containing the gene was sequenced and analyzed using NCBI’s BLAST (http://blast.ncbi.nlm.nih.gov/Blast.cgi) and in-house database.

Verified full length genes were sub-cloned into yeast expression vectors, pYES2.1 TOPO with URA as selection marker (Life Technologies, California) according to the protocol provided with minor modifications. The genes were transformed into a wild type yeast strains; BY4741 (ATCC No. 201388) and selected yeast mutant strains; TPI1^−^ (ATCC No. 4023986), FBA1^−^ (ATCC No. 4014909), GPD1^−^ (ATCC No. 4003718) and TDH3^−^ (ATCC No. 4004822) obtained from ATCC using lithium acetate transformation method. Empty vector was used as negative control. Selection of positive transformants was made on synthetic defined medium (without Uracil). For cross complementation study of glycolytic genes, *EgFBA* was transformed into yeast TPI knockdown strain, TPI1^−^.

### Verification of the yeast transformants

Analysis of the transformants was done by colony PCR using plasmid universal primer, GAL1 and gene specific reverse primers. Positive transformants were selected and analyzed for recombinant protein expression using Western Blot assay. Galactose was added as carbon source to induce protein expression in overexpressed and complemented yeast strains. Yeast cell lysate was harvested at several time points of 0, 6, and 8 hours. Total protein was extracted from the harvested cell lysate using TCA method^30^. Concentrations of protein were later determined using Bradford assay and diluted to 15 µg/ml before loaded on SDS-PAGE and run at 40 mA for 1 hour. Protein marker (PM) and Western marker (WM) were used as ladder for the assay. For the Western Blot assay, the SDS-PAGE was transferred onto a PVDF membrane at 150 mA for 2 hours. The membrane was blocked with 1x PBS with 0.1% Tween 20 for 40 minutes at room temperature, and washed three times before incubated with primary antibody overnight at 4 °C. Gene specific antibody; anti-TPI (1: 500), anti-FBA (1: 50000) and Anti-HisTag (1:1000) were used to detect the targeted protein. The membrane was washed three times before incubated with secondary antibody for 2 h. The membrane was later developed using BCIP/NBT color substrate (Promega Cooperation, Madison) for 5–20 minutes to observe the protein expression.

### Yeast growth conditions

Cells were grown in synthetic defined (SD) minimal agar medium supplemented with appropriate carbon source and auxotrophic supplements at 30 °C. Yeast strains were grown overnight in synthetic defined minimal liquid medium. Starter culture was prepared by inoculating overnight grown yeast culture with cell optical density at 600 nm (OD_600_) of 0.1 into fresh synthetic defined medium induced with galactose.

### Lipid analysis

Lipid quantification was performed using Nile red assay due to its throughput and relatively low sample volume requirement^31^. Yeast culture (1 mL) was harvested at late exponential growth phase (OD_600_ of 1.1) for total lipid content determination using Nile Red assay^32^. Cell density was adjusted to an optical density at OD_600_ of 0.5. For each strain, 250 µL of culture was transferred to a 96-well black microplate. Approximately, 25 µL of DMSO/medium (1:1, v/v) and 25 µL of 50 µg/mL Nile red are mixed into the culture for lipid content measurement. The fl uorescence intensity measurement with excitation and emission wavelength was set at 530 nm and 590 nm, respectively.

### Analysis of Single Nucleotide Polymorphism (SNP) effects and association data on glycolytic genes

Based on the verified oil palm glycolytic gene sequences, the data for the SNPs identified for each of the gene in this study was obtained as described by Teh *et al*.^33^. From the genotyping data, analysis was conducted on selected population of 576 palms from Nigerian x AVROS, 625 palms from Deli x AVROS and 675 palms from Deli x Dumpy AVROS. The association analysis was mainly focused on one of the yield component, oil to dry mesocarp content (O/DM). Each analysis was measured and analyzed with statistic tools; mean, T-test. For the combining ability analysis, the palms were segregated using Principle Component Analysis (PCA) into families with similar genotypes background. All the boxplots were generated using R software. Information on the SNPs location and prediction on the SNPs function is described in details in Supplementary Table 1 and 2.

## Electronic supplementary material

Supplementary Table 1

Supplementary Table 2

Supplementary Information
