# Supplementary material for: Key glycolytic branch influences mesocarp oil content in oil palm
Source: Sci Rep. 2017 Aug 29;7:9626. doi: 10.1038/s41598-017-10195-3 (PMC5575415; doi:10.1038/s41598-017-10195-3)
Supplement: Supplementary file 1 — Supplementary Table 1 [file 41598_2017_10195_MOESM1_ESM.pdf]

Key glycolytic branch influences mesocarp oil content in oil palm

Nurliyana Ruzlan[2], Yoke Sum Jaime Low[1], Wilonita Win[1], Noor Azizah Musa[1], Ai-Ling Ong[1], Fook-Tim Chew[3], David Appleton[1], Hirzun Mohd Yusof[2] & Harikrishna Kulaveerasingam[1]

[1]Biotechnology & Breeding Department, Sime Darby Plantation R&D Centre, Malaysia [2]Sime Darby Renewables, Sime Darby Plantation Sdn Bhd, Malaysia [3]Department of Biological Sciences, National University of Singapore, Singapore

Supplementary Table 1: List of SNPs identified on glycolytic genes with SNPs location information and function prediction.

SNP ID	Location / Position	Function prediction				Tag for Association study
		Transcription Factor binding site	Splicing potential (ESE/ESS)	Amino acid changes	microRNA binding site	
EgFBA						
SD_SNP_000007765	Intron/ 24881326	NA	Yes (218bp from Exon 3)	NA	NA	Yes Significant in Nigerian x AVROS
SD_SNP_000007766	3’ UTR/ 24879331	NA	NA	NA	No	Yes Significant in Deli x Dumpy AVROS
EgTPI						
SD_SNP_000151220	5’ UTR Promoter/ 19626161	No (1742bp from ATG)	NA	NA	NA	Yes Not significant in any cluster
SD_SNP_000035800	Intron/ 19621888	NA	No (>300bp from exon2)	NA	NA	Yes Not significant in any cluster
SD_SNP_000035801	Intron/ 19620304	NA	Yes (<300bp from exon 3)	NA	NA	Yes Significant in Nigerian x AVROS

***** NA refer to not available

SD_SNP_000035802	Intron/ 19619353	NA	Yes (<300bp from exon 4)	NA	NA	Yes<br>Significant in Nigerian x AVROS
SD_SNP_000035803	Intron/ 19615813	NA	Yes (>300bp from exon 9)	NA	NA	Yes<br>Not significant in any cluster
EgG3PDH						
SD_SNP_000008411*	5' UTR Promoter/<br>26031150	No (4707bp from ATG)	NA	NA	NA	Yes<br>Significant in Nigerian x PORIM & Deli x AVROS
EgGAPDH						
SD_SNP_000041010	3' UTR/ 21239379	NA	NA	NA	No	Yes<br>Not significant in any cluster
SD_SNP_000041011	Intron/<br>21235385	NA	Yes (118bp from exon 9)	NA	NA	Yes<br>Significant in Deli x Dumpy AVROS
SD_SNP_000041012	Intron/<br>21231353	NA	Yes (83bp from exon 3)	NA	NA	Yes<br>Significant in PORIM
SD_SNP_000041013	5' UTR Promoter/<br>21229798	No	NA	NA	NA	Yes<br>Not significant in cluster
