# Supplementary material for: Key glycolytic branch influences mesocarp oil content in oil palm
Source: Sci Rep. 2017 Aug 29;7:9626. doi: 10.1038/s41598-017-10195-3 (PMC5575415; doi:10.1038/s41598-017-10195-3)
Supplement: Supplementary file 2 — Supplementary Table 2 [file 41598_2017_10195_MOESM2_ESM.pdf]

Key glycolytic branch influences mesocarp oil content in oil palm

Nurliyana Ruzlan[2], Yoke Sum Jaime Low[1], Wilonita Win[1], Noor Azizah Musa[1], Ai-Ling Ong[1], Fook-Tim Chew[3], David Appleton[1], Hirzun Mohd Yusof[2] & Harikrishna Kulaveerasingam[1]

[1]Biotechnology & Breeding Department, Sime Darby Plantation R&D Centre, Malaysia [2]Sime Darby Renewables, Sime Darby Plantation Sdn Bhd, Malaysia [3]Department of Biological Sciences, National University of Singapore, Singapore

Supplementary Table 2: List of database use for SNP function prediction analysis

**Location of SNP**	**Online database**
SNP at promoter (5' UTR)	a) FAST-SNP – no longer available (http://fastsnp.ibms.sinica.edu.tw/pages/input_SNPListAnalysis.jsp)<br>b) LASAGNA-Search 2.0 (http://biogrid-head.engr.uconn.edu/lasagna_search/)<br>c) PLACE- Plant Cis-Acting Regulatory (http://www.dna.affrc.go.jp/PLACE/)
SNP at intron-exon junction	FAST-SNP – no longer available (http://fastsnp.ibms.sinica.edu.tw/pages/input_SNPListAnalysis.jsp)
SNP at exonic region	Amino acid changes analysis<br>Structure prediction analysis – SWISS-MODEL (http://swissmodel.expasy.org/)
SNP at 3' UTR	Oil palm genome browser (in-house database)
