# Supplementary material for: Key glycolytic branch influences mesocarp oil content in oil palm
Source: Sci Rep. 2017 Aug 29;7:9626. doi: 10.1038/s41598-017-10195-3 (PMC5575415; doi:10.1038/s41598-017-10195-3)
Supplement: Supplementary file 3 — Supplementary Information [file 41598_2017_10195_MOESM3_ESM.pdf]

Key glycolytic branch influences mesocarp oil content in oil palm

Nurliyana Ruzlan[2], Yoke Sum Jaime Low[1], Wilonita Win[1], Noor Azizah Musa[1], Ai-Ling Ong[1], Fook-Tim Chew[3], David Appleton[1], Hirzun Mohd Yusof[2] & Harikrishna Kulaveerasingam[1]

[1]Biotechnology & Breeding Department, Sime Darby Plantation R&D Centre, Malaysia [2]Sime Darby Renewables, Sime Darby Plantation Sdn Bhd, Malaysia [3]Department of Biological Sciences, National University of Singapore, Singapore

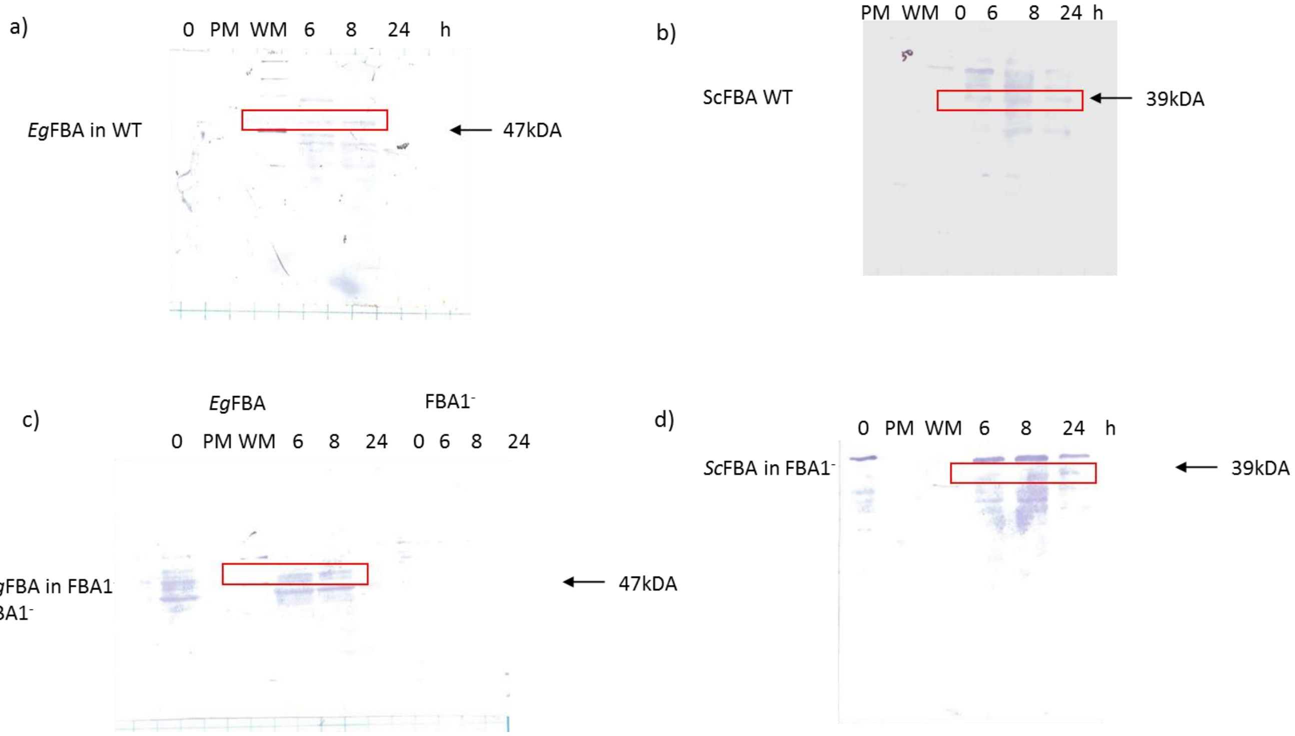


**Supplementary Figure 3**. **Expression of oil palm fructose-1,6-bisphophaste aldolase (*EgFBA*) using *Saccharomyces cerevisiae.*** Verification on *EgFBA* transformants was conducted using Western Blot assay with anti-FBA antibody (1:50000). (a) Protein of *EgFBA* was detected at 47kDa for overexpression of *EgFBA* in WT at 6 and 8 hours after induction with galactose. Lower size protein bands were observed at 0hr due to non-specific binding of the anti-FBA antibody. (b) *ScFBA* was also observed at 39kDa in the WT strain. (c) The function of *EgFBA* was validated as the protein band of *EgFBA* was observed in *EgFBA* complemented in FBA1$^-$ . This was further confirmed as no protein band was observed at 47kDa in the FBA1$^-$ strain. (d) Similarly, *ScFBA* band at 39kDa was also observed in the FBA1$^-$ indicating the presence of *ScFBA* in heterozygous knock out strain. PM and WM refer to protein marker and western marker used as ruler.

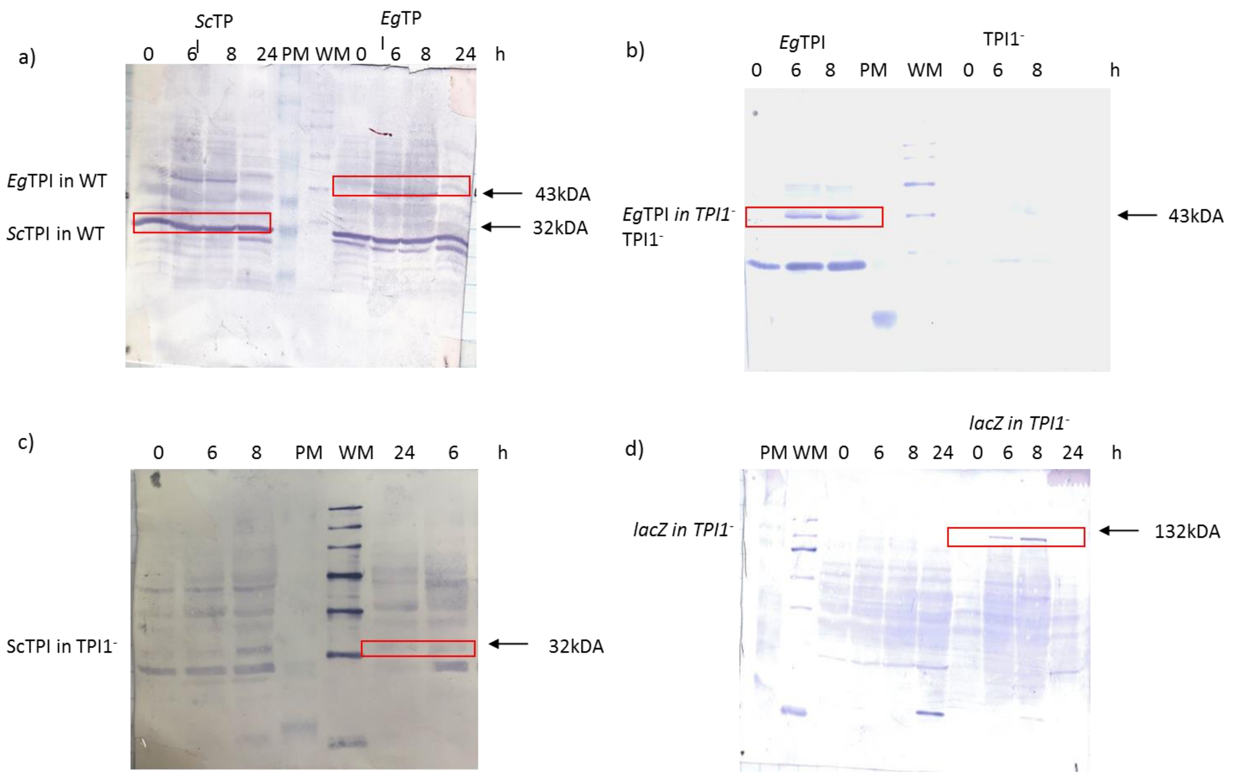


**Supplementary Figure 4**. **Expression of oil palm triose phosphate isomerase (*EgTPI*) using *Saccharomyces cerevisiae*** a) Protein of *EgTPI* was detected at 43kDa for overexpression of *EgTPI* in WT at 6 and 8 hours after induction with galactose. Lower size protein bands were observed due to non-specific binding of the anti-TPI antibody. *ScTPI* was also observed at 32kDa in the WT strain. b) The function of *EgTPI* was validated as the protein band of *EgTPI* was observed in *EgTPI* complemented in TPI1$^-$. This was further confirmed as no protein band was observed at 43kDa in the TPI1$^-$ strain. (c) Similarly, *ScTPI* band at 32kDa was also observed in the TPI1$^-$ indicating the presence of *ScTPI* in heterozygous knock out TPI1$^-$ strain. (d) lacZ in TPI1$^-$ serve as positive control for the assay. Presence of the 132Da band indicated presence of pYES 2.1 TOPO vector in TPI1$^-$. PM and WM refer to protein marker and western marker used as ruler.
